# Bladder Preservation with Concurrent Chemoradiotherapy Following Complete Response to Induction Systemic Therapy in Patients with Muscle-Invasive Bladder Cancer: A Review of the Existing Literature

**DOI:** 10.3390/cancers18060961

**Published:** 2026-03-16

**Authors:** Georgios Nikiforos Ntoumas, Andromachi Kougioumtzopoulou, Dimitra Desse, Charalambos Fragkoulis, Georgios Papadopoulos, Efthymios Kostouros, Dimitra Michaletou, Vassileios Kouloulias, Anna Zygogianni, Ioannis Georgakopoulos

**Affiliations:** 1 Department of Clinical Radiation Oncology, Medical School, ATTIKON University Hospital, National and Kapodistrian University of Athens, 124 62 Athens, Greece; ankougio@med.uoa.gr (A.K.); dimidesse@gmail.com (D.D.); demicha7@gmail.com (D.M.); vkouloul@med.uoa.gr (V.K.); azygogianni@med.uoa.gr (A.Z.); 2 Department of Urology, General Hospital of Athens “G. Gennimatas”, 115 27 Athens, Greece; harisfrag@yahoo.gr (C.F.); gipapadopoulos@yahoo.gr (G.P.); 3 3rd Department of Internal Medicine, General Hospital of Athens “G. Gennimatas”, 115 27 Athens, Greece; ekostouros@gmail.com; 4 Radiotherapy Unit, 1st Department of Radiology, Medical School, Aretaieion University Hospital, National and Kapodistrian University of Athens, 115 28 Athens, Greece; ioangeo@med.uoa.gr

**Keywords:** muscle-invasive bladder cancer, complete response, bladder preservation, chemoradiotherapy

## Abstract

Background/Objectives: The standard surgical treatment of muscle-invasive bladder cancer (MIBC) comprises neoadjuvant systemic therapy followed by radical cystectomy (RC). However, a notable number of patients achieve a favorable response to neoadjuvant systemic therapy, a finding associated with improved outcomes, and questioning the necessity of RC in this subset of patients. The objective of this review is to summarize the available evidence regarding the feasibility, efficacy and toxicity of bladder-preserving chemoradiotherapy (CRT) following clinical response (CR) to neoadjuvant systemic therapy in patients with MIBC. Methods: A literature search was performed using the PubMed database to identify studies evaluating the use of CRT for bladder preservation in MIBC patients with CR following neoadjuvant therapy. Results: Clinical complete response (cCR) rates to neoadjuvant systemic treatment ranged from 31% to 87.5%, with subsequent CRT enabling 50–97% of responders to retain their bladder. Long-term outcomes were favorable for cCR patients, with 3- to 5-year overall survival (OS) ranging from 65% to 89%, disease-free survival (DFS) of 64–86%, and bladder-intact survival up to 80%. Achievement of cCR, T2 tumor stage, absence of concomitant carcinoma in situ or hydronephrosis, and complete transurethral resection of the bladder tumor (TURBT) were prognostic factors for improved oncologic outcomes. Treatment-related toxicity was generally acceptable, concerning mainly hematological and gastrointestinal events, while severe late toxicity was uncommon. Conclusions: Bladder-preserving CRT is a promising, effective, and well-tolerated option for MIBC patients achieving CR to neoadjuvant systemic therapy. While further prospective validation and longer follow-up are required before it can universally replace radical cystectomy in this subset of patients, advances in neoadjuvant treatments, imaging and molecular biomarkers may improve response assessment and patient selection for bladder preservation.

## Simple Summary

Muscle-invasive bladder cancer (MIBC) mandates aggressive treatment with neoadjuvant systemic therapy followed by radical cystectomy for surgical candidates. However, a noteworthy subset of patients responds very well to initial therapy, and for these patients, it may be possible to treat MIBC with a combination of chemotherapy and radiotherapy, omitting cystectomy. Studies show this approach is generally safe, effective, and can preserve bladder function in selected patients. Improved induction treatments, advanced imaging techniques, and blood or urine biomarkers are awaited to better identify patients who can avoid cystectomy, without compromising oncological outcomes.

## 1. Introduction

Bladder cancer is the most prevalent cancer of the urinary tract, with an estimated incidence of over 600,000 new cases worldwide in 2022 [1]. In the United States alone, more than 84,000 new cases are diagnosed annually, making bladder cancer the fifth most common malignancy and the eighth leading cause of cancer-related death among men [2]. Muscle-invasion disease accounts for approximately one quarter of new diagnoses, and curative intent treatment typically involves either radical cystectomy (RC) or a trimodality (TMT) approach consisting of a maximal transurethral tumor resection followed by concurrent chemoradiotherapy (CRT) [3].

Before TMT emerged as an effective therapeutic alternative for selected patients, RC had long been considered the sole gold-standard management for muscle-invasive bladder cancer (MIBC) [4], providing a 5-year overall survival (OS) rate of approximately 50% [5,6,7]. However, this benefit has been achieved at the cost of substantial surgery-related morbidity and mortality as well as a significant impairment of quality of life [8,9].

The introduction of neoadjuvant cisplatin-based chemotherapy resulted in an absolute 5–8% improvement in 5-year OS, supported by randomized trials including SWOG 8710 and BA06 30894 and reinforced by individual patient data meta-analyses [10,11,12,13,14]. The VESPER (GETUG-AFU V05) trial subsequently provided contemporary evidence favoring dose-dense methotrexate, vinblastine, doxorubicin and cisplatin (MVAC) in the peri-operative setting, demonstrating improved progression-free survival (PFS) in patients treated with RC [15].

In addition, cisplatin-based neoadjuvant chemotherapy (NAC) is associated with substantial pathological downstaging at RC, achieving ypT0 rates of approximately 25–40% and ≤ypT2 rates of 45–75% across randomized trials and individual patient data meta-analyses, with the highest response rates (RRs) reported with dose-dense MVAC [10,12,13,14,15,16]. Current emerging data support the incorporation of perioperative immunotherapy in the NAC in this setting [17,18]. Specifically, immune checkpoint inhibition has been shown to enhance pathological response (PR), with durvalumab plus NAC achieving ypT0 rates approaching 40% and ≤ypT2 rates of approximately 70%, while enfortumab vedotin plus pembrolizumab yields ypT0 rates of around 30% in cisplatin-ineligible patients [17,18].

**Figure 1:**
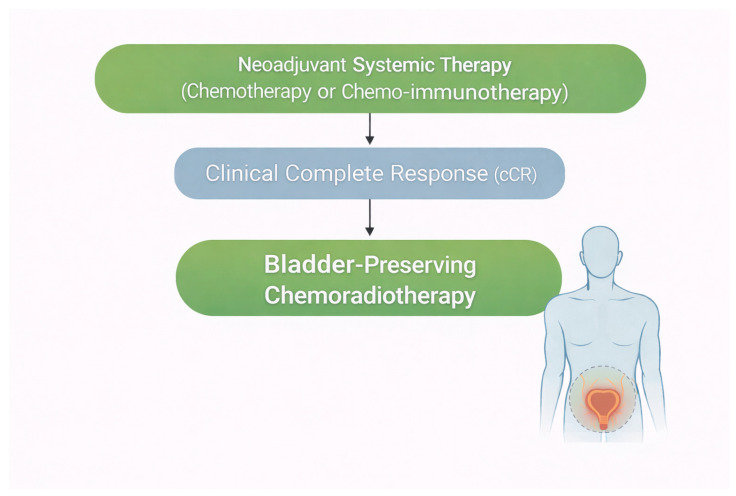
The concept of bladder preservation with chemoradiotherapy in patients with muscle-invasive bladder cancer achieving clinical complete response to neoadjuvant systemic therapy.

Achieving pathological downstaging to ypT0 or ≤ypT2 following induction systemic therapy has a profound impact on survival outcomes [19], raising concerns regarding the necessity of RC in this subset of patients and highlighting the need to investigate bladder-preserving strategies, including CRT [16]. The aim of this review is to summarize the existing literature on bladder preservation approaches using concurrent CRT as an alternative to RC in patients with MIBC who achieve a clinical response (CR) following neoadjuvant systemic therapy (Figure 1).

## 2. Materials and Methods

A comprehensive search of the PubMed database up to January 2026 was conducted to identify studies evaluating bladder-preserving CRT following clinical response to neoadjuvant systemic therapy in MIBC patients. Employed MeSH terms and keywords included “muscle-invasive bladder cancer”, “MIBC”, “urothelial carcinoma”, “neoadjuvant chemotherapy”, “neoadjuvant chemoimmunotherapy”, “induction chemotherapy”, “bladder preservation”, “bladder-sparing”, “trimodality therapy”, “chemoradiotherapy”, and “clinical complete response”. Boolean operators (AND/OR) were applied to combine search terms effectively and the following core search string was generated: (“muscle invasive bladder cancer” OR “bladder cancer” OR “urothelial cancer”) AND (“chemotherapy” OR “induction” OR “neoadjuvant chemotherapy” OR “neoadjuvant therapy” OR “neoadjuvant”) AND (“clinical complete response” OR “clinical response” or “response”) AND (“bladder preservation” OR “chemoradiotherapy” OR “bladder-sparing” OR “radiotherapy” OR “radiation”). Studies were eligible for inclusion if they reported RR, survival outcomes, bladder-intact survival, or treatment-related toxicity; studies lacking RR data for the applied treatment were excluded. Titles and abstracts identified through the search were screened for relevance. Full texts of potentially eligible articles were then reviewed to determine inclusion based on the predefined eligibility criteria. Additionally, reference lists of relevant articles were screened to identify further suitable studies. Only articles published in English were included and no filters regarding study design or country of origin were applied. From each included study, the following data were extracted: study design and publication year, patient population and sample size, type of induction or neoadjuvant therapy, definition and assessment of clinical response, details regarding the chemoradiotherapy bladder-preserving regimen, oncologic outcomes including response to applied neoadjuvant/induction treatment, bladder preservation rates and reported treatment-induced toxicity. We also browsed the Clinical Trials website to gain access to ongoing trials examining relevant issues.

Given the heterogeneity of the published literature, which included both retrospective and early-phase prospective series, a formal quantitative risk-of-bias assessment was not conducted. Instead, studies were qualitatively assessed with emphasis on study design, sample size, and completeness of reported outcomes.

**Figure 2:**
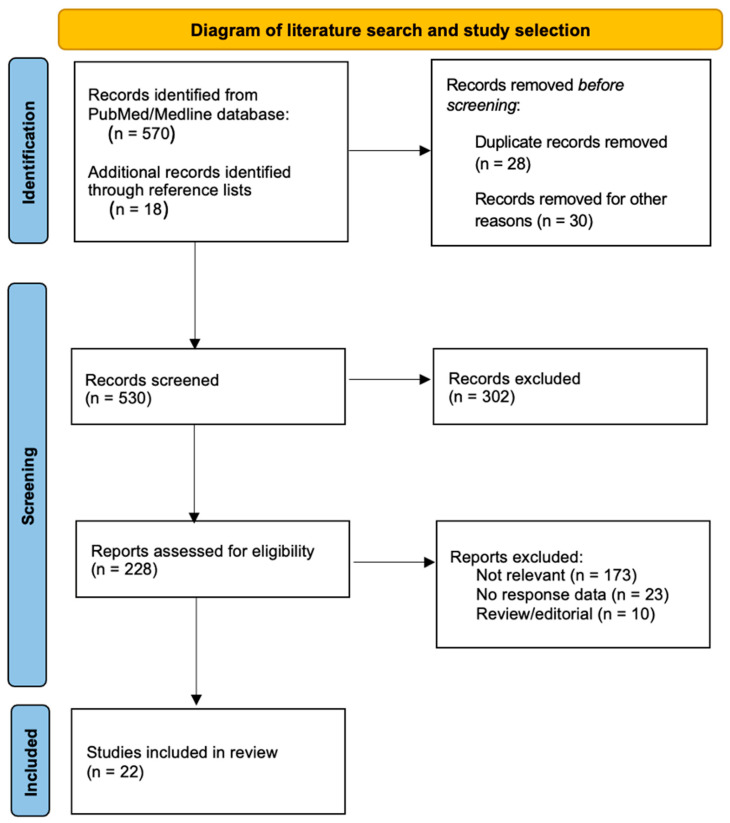
Flow diagram of study identification and selection (adapted from the PRISMA 2020 framework).

A flow-diagram of literature search and study selection adapted from the PRISMA 2020 framework is illustrated in Figure 2.

## 3. Results

We found 22 studies that have attempted to investigate the feasibility, efficacy and toxicity of replacing RC with CRT in the treatment of patients with bladder cancer, achieving CR to neoadjuvant systemic therapy.

### 3.1. Efficacy

Early data on the efficacy of NAC followed by concurrent CRT for MIBC were derived from studies conducted during the late 20th century, attempting to implement induction chemotherapy as a means to select patients for bladder preservation (Table 1). Such prospective trials incorporating transurethral resection of bladder tumour (TURBT), NAC with methotrexate, vinblastine, cisplatin with or without doxorubicin, and subsequent CRT in responders reported overall RR to NAC ranging from 53% to 100%, with 31% to 87.5% of patients achieving clinical complete response (cCR) [11,20,21,22,23,24,25,26,27].

After median follow-ups of 23 and 31 months, Červek et al. [22] and Danesi et al. [25] reported similar results among complete responders to NAC receiving either subsequent radiotherapy or CRT with cisplatin/5FU, respectively. Among the 24 and 21 complete responders included in the two studies, OS rates were 89% and 86%, while bladder tumour-free survival rates were 78% and 71.4%, respectively [22,25]. Salvage RC rates were 6.4% and 14.2%, respectively, both lower compared to the one mentioned by Vogelzang et al. [21,22,25]. The study by Červek et al. further compared complete and non-complete responders to NAC, with the former showing significantly higher OS (89 vs. 49%, *p* = 0.0025) and lower distant metastases rate (10 vs. 57%) [22].

On the other hand, the findings by Given et al. [24] were not as favorable as those of the above series. Ninety-four patients with MIBC who were either of increased surgical risk or who declined surgery all underwent neoadjuvant cisplatin-based chemotherapy (MVAC or methotrexate, vinblastine and cisplatin-MCV). Thirty-three responders at post-NAC restaging (40% CR and 19% PR) received concurrent CRT with weekly cisplatin at a total dose of 64.8 Gy, while no responders continued with surveillance, partial or radical cystectomy. Twenty-two percent of CRT-treated patients had no response or progressed, 8.2% died and 14.3% required salvage RC, whereas at 5 years, the absolute survival rate for the same patient group was diminished to 39%, compared to 43%, 55% and 71% for those undergoing surveillance only, partial and radical cystectomy, respectively. Eventually, the 5-year bladder preservation rate was 53%, with only 18% of patients remaining alive with an intact bladder across the entire study cohort. Regarding relapse-free survival rates, a statistically significant difference of 40% vs. 65% (*p* = 0.009) was illustrated between patients preserving their bladder and those having it removed, respectively [24].

The Radiation Therapy Oncology Group (RTOG) has also conducted trials to examine the role of NAC combined with bladder preservation with CRT for responding patients [20,26]. Beginning with the phase II RTOG 88-02 trial, 91 patients with T2-T4aM0 bladder cancer who were candidates for RC were treated with two cycles of neoadjuvant methotrexate, cisplatin and vinblastine, followed by an “induction” CRT regimen with concurrent cisplatin-radiotherapy to a dose of 39.6 Gy. Complete responders, defined by negative examination under anesthesia, cystoscopy with biopsy, urine cytology and pelvic computed tomography scan, proceeded to a consolidative cisplatin-radiotherapy course to a total dose of 64.8 Gy, while non-responders underwent immediate RC. Overall, 68 of 85 evaluated patients (80%) achieved a CR to induction CRT, and 70 patients proceeded to consolidation CRT, of whom 50 patients preserved their bladder during follow-up. Ultimately, the 4-year actuarial overall- and bladder-intact-survival for the whole series were 62% and 44%, respectively [20].

To further elucidate the potential favorable effect on oncologic outcomes with the addition of NAC prior to CRT for the treatment of MIBC, the RTOG conducted the 89-03 trial, a phase 3 randomized trial that followed the same treatment protocol as the RTOG 88-02 trial but additionally incorporated a comparator arm where no NAC was administered. Despite 60.3% of 58 restaged patients experiencing CR to NAC and induction CRT, after a median follow-up of 60 months, the trial concluded that NAC with two cycles of MCV prior to split-course CRT demonstrated no improvement in CR, survival with a functioning bladder, OS and metastases-free survival rates [26].

More contemporary series on the issue of CRT after response to NAC showed promising results regarding survival and bladder preservation rates [28,29,30,31,32,33,34,35,36,37,38,39,40,41].

Four prospective trials included MIBC patients with T2-T4 and T2-T3N0M0 disease and implemented induction chemotherapy with two or three cycles of CMV, MVAC, gemcitabine-cisplatin or cisplatin-5-fluorouracil with or without paclitaxel [30,32,34,35]. The entire study population ranged from 29 to 104 patients. Similar to the RTOG’s protocols, the trials followed a response-guided treatment approach using interval cystoscopy, imaging and/or tumour site biopsy and incorporated split-course cisplatin-based CRT up to a total dose of 60–65 Gy for complete responders to induction chemotherapy and to subsequent CRT. CR was accomplished in 68% to 86% of patients, corresponding to 34/50, 82/104, 25/29, and 23/30 patients, across the four studies [30,32,34,35]. Reported 5y-OS rates ranged from 48% to 72% for the entire study population [30,32,34,35] and the median intact-bladder survival rate reached approximately 80% in one series [34]. Among the 82 and 34 complete responders to NAC in the studies by Sabaa et al. and Arias et al., respectively, 5y-OS, DSS and LC rate were 65–67.6%, 75.7% and 70%, respectively [30,35]. In the former study, superficial recurrences and distant metastases occurred in 8.1% and 24.3% of complete responders, respectively [35].

Other recent prospective studies employed NAC followed by continuous-course radiotherapy with or without concurrent chemotherapy, with treatment response assessed prior to completion of NAC [36], at the interval between NAC and radiotherapy [37,38,39] or after completion of radiotherapy [33]. In two phase II trials and two prospective trials, patients with MIBC-staged T2-T4N0M0 were treated with modern neoadjuvant regimens consisting of three to four cycles of gemcitabine-cisplatin [36,37,38,39]. Assessment of response to NAC by Dracham et al. and Agrawal et al. was based on RECIST criteria, and most of the patients with at least 50% or more tumour response or tumour downstaging to ≤T1 at repeat cystoscopy/TURBT received radiotherapy (60–66 Gy) with or without cisplatin [36,37,38,39]. Notably, in the trial by Shi et al., patients underwent multiparametric MRI, apart from cystoscopy and urine cytology, for further clinical staging of response [36]. RR to NAC ranged from 53% to 87.5%, corresponding to 31/59, 27/34, 25/30, and 35/40 patients, across the four trials [36,37,38,39]. After a median follow-up of over 40 months in both phase II trials, oncologic outcomes were favorable for the bladder-sparing group and especially for definite responders (CR or ≤T1), with 3-year OS, DFS and RFS rates of nearly 89%, 85.7% and 74.3%, respectively, vs. 40–60%, 26.7% and 37.5%, respectively, in the non-responding/RC group [36,38]. When patients with CR and PR > 50% were compared in the studies by Dracham et al. and Elsayed et al., the former subgroup reached superior 3y-OS (85.7–89% vs. 73.1–75%, *p* = 0.06 and 0.001), DFS (76–85.7% vs. 40–40.4%, *p* = 0.02 and 0.013) and MFS (87.9 vs. 51.3%) rates [38,39]. Shi et al. further demonstrated that 97.1% (34/35) of patients treated with bladder sparing approaches (80% receiving CRT) continued retaining their bladder, and 85.7% had remained tumour-free by the last follow-up [36].

Finally, in the evolving landscape of neoadjuvant systemic therapy with the incorporation of immunotherapy, there is growing interest in bladder-preserving strategies for patients with CR after induction chemo-immunotherapy. In the post hoc analysis of two prospective studies of neoadjuvant cisplatin-based chemotherapy followed by selective CRT for MIBC-staged cT2-T4aN0M0, approximately 16% of patients received gemcitabine-cisplatin plus nivolumab; however, this subgroup was not analyzed separately in the study. CR was achieved in 66 of 76 patients (86.8%), and all 76 patients received CRT instead of RC. After a median follow-up of 64 months, salvage RC and recurrence rate were 13.1% and 57%, respectively, while DFS for the entire study population was 46.3 months, and median MFS was not reached. Among 66 patients with cCR after neoadjuvant systemic therapy, 5 y DFS and MFS rates were 38% and 70%, respectively, with DFS being significantly longer compared with patients without CR (hazard ratio, 0.465; 95% CI, 0.222 to 0.976) [40].

In a recently published phase II study, investigators broadened their inclusion criteria by enrolling 45 patients considered non-ideal candidates for bladder-preserving strategies, with high-risk, locally advanced MIBC of stage cT2-T4bN0–3M0–1a. Patients who showed no disease progression after two cycles of neoadjuvant gemcitabine-cisplatin plus anti-PD1 agent tislelizumab completed neoadjuvant treatment with one or two additional cycles, resulting in a CR rate of 51.11%. After neoadjuvant treatment, 36 patients underwent radiotherapy and consolidation tislelizumab for up to one year, leading to CR (post-radiotherapy Tis or T0) in 33 of 36 patients. Three-year PFS, bladder-intact-DFS, and OS were 76%, 76% and 81%, respectively. The authors concluded that these findings could support the use of neoadjuvant chemo-immunotherapy to successfully render locally advanced MIBC patients—an otherwise suboptimal patient group for omission of RC—suitable for bladder preservation, with favorable long-term oncologic outcomes [41].

**Table 1:** Key studies evaluating bladder-preserving chemoradiotherapy (CRT) following response to neoadjuvant systemic therapy in patients with muscle-invasive bladder cancer (MIBC).

Study	Study Design	Patient Population	No of Patients	Follow-Up	Neoadjuvant Regimen	cCR Definition	RT/CRT Approach	Results
Vogelzang et al. [21] 1993	Prospective	Τ2–Τ4Ν0–Ν2	29	57 mo	MVAC 4 cycles	negative physical examination, cystoscopy, biopsy, urine cytology, CT, bone scan, chest radiograph	RT (66 Gy) after cCR	Response rate 69% (31% cCR and 38% cPR) 14/15 patients treated with RT disease-free; salvage RC in 5 pts
Červek et al. [22] 1993	Prospective	T2–T4NxM0	47	23 mo	MCV 3–4 cycles	negative cystoscopy, biopsy & urine cytology	RT (64–66 Gy to bladder and 40 Gy to PLNs) after cCR	cCR 53% bladder intact and tumor–free 78% OS 89% in CR vs. 49% (*p* = 0.0025); DM 10% in CR vs. 57% in non-CR
Housset et al. [23] 1993	Prospective	T2–T4	44	27 mo	5FU/cisplatin + RT (24 Gy)	negative cystoscopy and deep biopsies ± urine cytology	RT boost (total 44 Gy) + 5FU/cisplatin after cCR	cCR 74% 3y-DFS 77% in CR vs. 23% in non-CR (*p* = 0.001) 3y-OS 81% in CR
Given et al. [24] 1995	Prospective	T2–T4NXM0	94	>5 y	MVAC or MCV 2–3 cycles	negative radiographic, cystoscopic & pathologic evaluation	CRT (weekly cisplatin, 64.8 Gy) after cCR or cPR	Response rate 59% (40% cCR and 19% cPR) 5y OS 39% in RT-treated; 18% alive with intact bladder by last fup
RTOG 88–02 [20] 1996	Phase II	T2–T4aN0M0	91	3.8 y	MCV (2 cycles)	negative physical examination, cystoscopy, biopsy, urinary cytology, pelvic CT scan	Split-course CRT (64.8 Gy, cisplatin)	cCR 80%; 4y-BP and OS 44% and 62%, respectively (for whole series)
Kachnic et al. [27] 1997	Prospective	T2–T4aNxM0	106	4.4 y	MCV 2 cycles	negative cystoscopy, biopsy, urine cytology	Split-course CRT (64.8 Gy total, cisplatin); total dose for cCR after induction CRT	cCR 66%; 5y-OS with BP 43%
Danesi et al. [25] 1997	Prospective	T2–T4aNXM0	25	31 mo	MCV 2 cycles	negative CT scan and TURBT	CRT (70 Gy, cisplatin/5-FU) after cCR	cCR 87.5% OS 86% in CR 71.4% tumor–free bladder
RTOG 89–03 [26] 1998	Phase III—randomised	T2–T4aN0M0	123; 61 received NAC (arm 1)	60 mo	MCV vs. none	negative physical examination, cystoscopy, biopsy, urinary cytology, pelvic CT scan	Split-course CRT (64.8 Gy total, cisplatin); total dose for cCR after induction CRT	5y-OS 48% arm 1 vs. 49% arm 2 (NS); 5y-DMR 33% arm 1 vs. 39% arm 2 (NS); alive + functionning bladder at 5 y 36% arm 1 vs. 40% arm 2 (NS)
Arias et al. [30] 2000	Prospective	T2–T4	50	73 mo	MVAC 2 cycles	negative physical examination, biopsy, urine cytology	Split-course CRT (65 Gy total to bladder + PLNs, cisplatin); total dose for cCR after induction CRT	cCR 68% 5y-OS 65% in cCR; 5y-LC 70% in cCR
George et al. [31] 2004	Retrospective	T2–T4N0–N1M0	60; 22 patients received NAC	48.5 mo	MVAC or MCV 2–4 cycles	negative cystoscopy and biopsy	CRT (65 Gy total to bladder + PLNs, cisplatin or carboplatin or cisplatin/5FU)	cCR 55% 5y-OS 36% (whole series) NAC and cCR NS for survival on univariate analysis
Cobo et al. [32] 2006	prospective	T2–T3NxM0	29	69.4 mo	CMV or GC 2 cycles	negative biopsy	Split-course CRT (64.8 Gy total, cisplatin); total dose for cCR after induction CRT	cCR 86%; 48% of *p* alive + intact bladder
Perdonà et al. [33] 2008	prospective	T2–T4	121	66 mo	MCV	negative cystoscopy, bipsy, urine cytology	RT (43p, 65 Gy total to bladder + PLNs) or CRT (78p, cisplatin); for all p	cCR 85.7% 5y-DSS, OS, and BIS 73.5%, 67.7%, and 51.2%, respectively
Lin et al. [34] 2009	prospective	T2–T4aN0M0	30	47 mo	CF or paclitaxel-CF 3 cycles	negative cystoscopy, biopsy, urine cytology	CRT (64.8 Gy total, cisplatin or CF) after cCR	cCR 73%; 3y-OS and PFS 77% and 54%, respectively (whole series); 63.6% RFS in cCR
Sabaa et al. [35] 2010	Prospective	T2–T3aN0M0	104	71 mo	GC 3 cycles	negative abdominoplevic CT, cystoscopy, biopsy	Split-course CRT (60–65 Gy total to bladder and PLNs, cisplatin); total dose for cCR after induction CRT	cCR 78.8%; 5y-OS 67.6% in cCR; 5y-DSS 75.7% in cCR
BA06 30894 [11] 2011	Phase III—randomised	T2–T4aNxM0	976; 485 no NAC vs. 491 NAC	8 y	MCV (3 cycles)	NR	RT (49%) or RC regardless of cCR (not assessed)	CMV resulted in 20% reduction in the risk of death (*p* = 0.070) and 9% reduction in locoregional failure (*p* = 0.417)
Araujo et al. [28] 2015	retrospective	cT2–T4 N0–N2 M0	22	24 mo	platinum-based (64% GC)	negative abdominopelvic CT, cystoscopy +/− biopsy	CRT (63 Gy median total dose to bladder + PLNs, platinum or gemcitabine) for responders or SD	cCR 68.2%; 3y-LC 90.9% in cCR 3y-OS 64.6% in cCR vs. 57.1% in cPR (*p* = 0.046); 3y-DFS 64.3% in cCR vs. 57.1% in cPR (*p* = 0.03);
Jiang et al. [29] 2019	Retrospective	T2–T4N1–N2	57	19.3 mo	GC 2–4 cycles	negative cystoscopy or imaging	CRT (60–66 Gy total to bladder + PLNs, cisplatin) for responders or SD	ORR (based on imaging) to NAC 56% (19% cCR, 38% cPR); 41% SD; 70% cCR in postNAC cystoscopy; 2y-OS 74%; 2y-DSS 88.3%; 2y-BI-DFS 64.2%; salvage RC 14%
Shi et al. [36] 2021	Phase II	T2–T4aN0M0	59	44.6 mo	GC 2–4 cycles	negative imaging, cystoscopy, biopsy	CRT (65 Gy total to bladder + PLNs, cisplatin) for definite responders (≤T1)	Response rate (≤T1) 53%; 3y-OS: 88.4% and 3 y RFS: 74.3% for BP group; 97.1% bladder-intact and 85.7% tumor-free by last fup
Agrawal et al. [37] 2020	prospective	T2–T4	30	NR	GC or gem-carbo 3 cycles	negative cystoscopy, imaging	CRT (60 Gy total to bladder + PLNs, cisplatin) for responders (RECIST 1.1)	Response rate 83.33% (cCR 56.67% and cPR 26.66%)
Dracham et al. [38] 2022	Phase II	T2–T4aN0M0	40	43 mo	GC 3 cycles	RECIST criteria	RT (2/35 *p*) or CRT (33/35 *p*) (66 Gy to bladder + PLNs, cisplatin) after cCR or cPR ≥ 50%	Response rate 87.5% (22.5% CR and 65% PR ≥ 50%); 3y-OS 89% in cCR vs. 73.1% in cPR ≥ 50%; 3y-DFS 85.7% in cCR vs. 40.4% in cPR ≥ 50%; 3-year MFS 87.9% in cCR vs. 51.3% in cPR ≥ 50%
Elsayed et al. [39] 2023	prospective	T2–T4aN0M0	34	46 mo	GC 3 cycles	RECIST criteria	CRT (64 Gy total to bladder + PLNs, cisplatin) for cCR or cPR ≥ 50%	Response rate 79.4% (61.7% CR and 17.6% PR > 50%); LRFS 75.1%; DMFS 77.8%; 3y-OS 85.7% in cCR vs. 75% in cPR > 50%; 3y-DFS 76% in cCR vs. 40% in cPR > 50%;
Cho et al. [40] 2024	Post Hoc of 2 phase II trials	T2–T4aN0M0	76	64 mo	GC + nivolumab (subset)	negative imaging, cystoscopy, cytology	CRT (up to 70.2Gy total, cisplatin) after cCR	cCR 86.8% 5y-DFS 38%; 5y-MFS 70%; salvage RC 13.1%
Wen et al. (HOPE-02) [41] 2025	Phase II	cT2–T4bN0–3M0–1a	45	36.1 mo	GC or gem-carbo 3–4 cycles + tislelizumab	NR	RT (60.4–64.4 Gy to bladder + PLNs) + consolidation tislelizumab for non-PD *p*	response after RT in 33/36 *p*; 3y-OS 81%; 3y-BI-DFS 76%;

Abbreviations: BP, bladder preservation; BI-DFS, bladder-intact disease-free survival; BIS, bladder-intact survival; cCR, clinical complete response; cPR, clinical partial response; CF, cisplatin and 5-fluorouracil; CMV/MCV, cisplatin, methotrexate, and vinblastine; CRT, chemoradiotherapy; CT, computed tomography; DFS, disease-free survival; DM, distant metastasis; DMFS, distant metastasis-free survival; DMR, distant metastasis rate; DSS, disease-specific survival; fup, follow-up; GC, gemcitabine and cisplatin; gem-carbo, gemcitabine and carboplatin; Gy, gray; LC, local control; LRFS, locoregional recurrence-free survival; MFS, metastasis-free survival; mo, months; MVAC, methotrexate, vinblastine, doxorubicin, and cisplatin; NAC, neoadjuvant chemotherapy; NR, not reported; NS, not significant; ORR, objective response rate; OS, overall survival; p, patients; PFS, progression-free survival; PLNs, pelvic lymph nodes; PR ≥ 50%, partial response of at least 50%; RC, radical cystectomy; RECIST, Response Evaluation Criteria in Solid Tumors; RFS, recurrence-free survival; RT, radiotherapy; SD, stable disease; y, years.

### 3.2. Prognostic Factors Associated with Clinical Response and Various Survival Outcomes

One of the earliest analyses to evaluate clinical and pathological factors associated with improved outcomes in patients with MIBC treated with NAC followed by CRT for complete responders demonstrated that T2 tumour stage and the absence of concomitant carcinoma in situ were significant predictors of CR. The rate of distant metastases was correlated with response to CRT as well as tumour stage and tumour size [42].

Most of the aforementioned studies further incorporated univariate and multivariate analyses to identify prognostic factors associated with CR and other oncologic outcomes. In accordance with the findings reported by Fung et al., T2 disease was consistently shown in multiple studies to be significantly associated with higher CR rates [24,33,35,40], improved local control [38], relapse-free survival, disease-free survival, OS, and bladder-intact survival compared with more advanced tumour stages [28,31,32,33,35,38,40]. Nevertheless, some trials failed to confirm a significant association between the T2 stage and either RR or survival outcomes [20,26,34].

The presence of hydronephrosis was significantly associated with an increased risk of distant metastases and mortality in both the RTOG 8802 and 8903 trials and one retrospective trial, and was additionally linked to a reduced CR rate in RTOG 8903 [20,26,29]. Moreover, complete TURBT was identified in several studies as a significant predictor of CR, bladder-intact survival, and OS [26,32,33].

Regarding tumour grade, Sabaa et al. [35] reported that it was the most significant predictor of CR on both univariate and multivariate analyses. Tumour grade also independently predicted the occurrence of distant metastases, together with tumour stage, tumour size, and multifocality. In a separate analysis restricted to patients achieving a CR following induction CRT, age, tumour number, grade, and stage were identified as significant predictors of OS [35]. Advanced age (≥70 years) was additionally associated with inferior progression-free survival [34].

Focusing on the prognostic significance of cCR, Cho et al. demonstrated that achievement of a cCR was associated with improved disease-free survival (DFS) [40]. Jiang et al. observed that residual disease on post-NAC cystoscopy predicted inferior BI-DFS [29]. The predictive value of cCR was further corroborated by the multivariate analysis and stratification performed by Dracham et al. and Araujo et al., respectively, which both confirmed a statistically significant association between cCR and improved local control, disease-free survival, and OS [28,38].

Conversely, George et al. failed to prove an association between NAC before CRT and cCR on post NAC cystoscopy with increased survival outcomes [31].

The absence of benefit from the addition of NAC for patients treated with radiotherapy instead of RC was also noted in the long-term results of the international multicenter phase III randomized trial BA06 30894, even though NAC RR was not part of its endpoints. In total, 976 patients were randomly assigned either to receive or omit three cycles of neoadjuvant CMV before radical local treatment (radiotherapy or RC). Overall, 43% of patients opted for radiotherapy. After a median follow-up of 8 years, the findings did not reach statistical significance in the radiotherapy group. More specifically, MCV resulted in a 20% reduction in the risk of death (HR, 0.80; 95% CI, 0.63 to 1.02; *p* = 0.070) and a 9% reduction in locoregional failure (HR, 0.91; 95% CI, 0.73 to 1.14; *p* = 0.417) [11].

In accordance with the previous results, the pooled analysis of several Massachusetts General Hospital (MGH) and RTOG trials failed to prove a correlation between NAC prior to bladder-preserving CRT and enhanced OS or DSS rates [43].

### 3.3. Toxicity

Combined modality treatment comprising NAC followed by CRT was well-tolerated across most of the aforementioned series.

Most cohorts receiving NAC with MCV regimen with or without doxorubicin followed by split-course or continuous CRT reported mainly grade 1–2 acute hematological, gastrointestinal and urinary toxicity, with the most common events being nausea, vomiting, diarrhea, rectal tenesmus, urinary frequency, dysuria, leukopenia, anemia and thrombocytopenia [20,21,22,25,32]. Reported acute grade 3–4 leukopenia, thrombocytopenia, bowel and bladder toxicity ranged from 8 to 26%, 2 to 24%, 1 to 12.4% and 3–11.5%, respectively [20,21,22,25,32]. Nausea and vomiting were mainly attributed to MCV chemotherapy, whereas CRT contributed to bladder and bowel toxicity [21,32]. With respect to late toxicity, most symptoms were mild and either improved or completely resolved within weeks after treatment completion [22,33]. Only a small number of patients required salvage RC due to treatment-related adverse events, including one patient with dystrophic calcification reported by Vogelzang et al. and one patient with a contracted bladder reported by Perdonà et al. [21,33]. Similarly, surgical intervention for late grade 4 gastrointestinal toxicity was rarely required. Moreover, in the study by Perdonà et al., approximately 6% of patients with preserved bladder suffered from reduced bladder capacity post-radiotherapy, while late mild dysuria, diarrhea, urinary urgency and nocturia were observed in 9 to 24% of patients [33].

In contrast to the above studies, RTOG 89–03 reported an unacceptably high rate of acute grade 3–4 toxicity in the arm receiving neoadjuvant MCV compared to the control arm, with 23% of patients experiencing neutropenic fever and five patients dying from sepsis and gastrointestinal bleeding. In addition, a relatively high persistence of severe late toxicity was noted, including grade 3–4 hematologic and bladder adverse events in 13% and renal and gastrointestinal toxicity in 8% of patients. This unexpected high severe toxicity occurrence with the addition of neoadjuvant MCV, as well as the poor patient accrual rate, led to the premature cessation of the trial [26].

More recent studies incorporating neoadjuvant gemcitabine and cisplatin instead of MCV have demonstrated more favorable toxicity profiles. Cobo et al. [32] found reduced rates of acute toxicity with neoadjuvant gemcitabine–cisplatin, with grade 3–4 neutropenia, febrile neutropenia, and thrombocytopenia observed in 7% of patients, compared with corresponding rates of 26%, 20%, and 13%, respectively, among patients receiving MCV [32]. In studies solely employing GC as induction chemotherapy, low rates of severe gastrointestinal toxicity were reported (1.7–5.7%), along with grade 3–4 myelosuppression in 5.7–21% of patients [29,35,36,37,38]. Sabaa et al. and Shi et al. reported no grade 3 adverse events attributable to concurrent CRT, except for radiotherapy-related skin toxicity, which occurred in 3.8% of patients in the former study [35,36]. As far as late toxicity is concerned, no cases of radiotherapy-induced contracted bladder or of late gastrointestinal toxicity occurred [35,36,38]. Shi et al. further included an analysis on functional outcomes at 6 months and confirmed significantly superior results regarding both total physical and mental health scores for the bladder-sparing group compared to the cystectomized group [36].

Lastly, studies implementing neoadjuvant chemo-immunotherapy with gemcitabine-cisplatin and either nivolumab or tislelizumab followed by bladder preserving CRT, reported generally acceptable toxicity rates [40,41]. CRT-induced intractable bleeding and recurrent urinary tract infections were noted in 10.5% of patients [40].

## 4. Discussion

This review explored the published literature regarding the feasibility, efficacy and toxicity of applying NAC followed by bladder preservation CRT in responding patients, as well as prognostic factors associated with improved outcomes when such an approach is selected. Despite the inevitable heterogeneity in protocols and methods of included studies—attributed not only to institutional differences but also to evolutionary changes in oncological practices over time—the reported results are generally satisfactory.

cCR rates to NAC ranged widely from 31 to 87.5% [11,20,21,22,23,24,25,26,27], with more contemporary trials demonstrating rates above 66% [30,32,34,35]. Bladder preservation was ultimately achieved in 44 to 97% of patients, and 5-year OS rates reached 39 to 72% [30,32,34,35]. The observed variability in the rates can be partly explained by differences in patient selection, chemotherapy and radiotherapy regimens, as well as response assessment methods. For example, while most of the trials included patients with T2-T4N0M0 MIBC [20,21,22,23,24,25,26,27,30,31,33,34,36,38,40], some protocols excluded T4 tumors [32,35], whereas others extended the criteria to include patients with positive regional nodes or even M1a disease [21,41]. Additionally, older trials incorporated NAC with MCV [20,21,22,24,25,26,31,32,33], a regimen that is no longer recommended in this context in current international oncology guidelines [44,45]. Radiotherapy approaches varied substantially across trials, both in terms of radiotherapy scheduling (split vs. continuous course) and the techniques employed, with more conformal radiotherapy planning being available and applied in more contemporary series [28,36,38,40]. Various authors followed different approaches in defining response to NAC and in timing of assessment; most studies incorporated pathologic confirmation in addition to cystoscopy, cytology and CT scan [20,22,24,32,33,34,35,38], while more recent studies further introduced magnetic resonance imaging (MRI) or multiparametric MRI in response assessment [36,38]. Interestingly, Jiang et al. observed a significant discrepancy between post-NAC imaging and cystoscopy in identifying CR and PR, further highlighting the potential variability associated with the implementation of different modalities for post-NAC response evaluation [29]. With respect to timing, patients were either assessed after completion of NAC [22,36,38] or in between induction and consolidation CRT in the case of split-course scheduling [20,26,27,32,35]. Lastly, several protocols mandated a CR to induction chemotherapy or CRT for patients to proceed to bladder preservation [20,26,27,30,32,34,35], while others expanded their criteria allowing partially responding patients and even patients with stable disease to omit RC [24,29,36,38,39,40,41]. Consequently, the currently available evidence should be interpreted cautiously, as the heterogeneity in study design, response assessment, and treatment protocols limits the ability to draw definitive conclusions regarding the optimal bladder-preserving strategy.

Historically, RC has long been the gold standard for treating MIBC patients, with bladder preservation reserved solely for patients who either declined surgery or who were medically unfit for it [46]. Despite the absence of a head-to-head, randomized comparison of RC with bladder-preserving combined modality treatment, recent data from high-quality, large retrospective comparative studies established the equivalent efficacy of both approaches [47,48], with current international guidelines incorporating them both as equal treatment choices for T2-T4aN0 and even N1 MIBC patients [45]. Of course, disease features including T2 stage, unifocality, absence of hydronephrosis and of diffuse CIS and tumour amenability to complete TURBT are well-established characteristics to define ideal candidates for bladder preservation strategies [46,49]. The significance of these features as predictors of cCR post-NAC and improved outcomes in patients treated with CRT after CR was also confirmed in our review.

With the introduction of NAC, its progressive improvement over time and recent updates following approval of perioperative chemoimmunotherapy, the emerging clinical subgroup of complete responders becomes increasingly apparent as potential candidates for bladder-sparing approaches [16,17,18,50]. Recent studies have estimated that 30–40% of patients achieve pathologic complete response (pCR) following neoadjuvant systemic treatment and RC, reaching a 5-year OS of up to 80% [16]. If cCR is considered an acceptable surrogate for pCR and given that up to 85% of otherwise surgical candidates experiencing cCR refuse RC and elect bladder preservation, it is unsurprising that bladder preservation strategies, including CRT, in this patient subgroup have attracted substantial interest [16,50,51].

Nevertheless, the above approach raises several concerns. First, despite the rising interest in replacing RC, bladder preservation approaches following cCR to neoadjuvant systemic treatment remain investigational, as emphasized in a recently published article [50]. The feasibility and safety of replacing RC with CRT have been demonstrated in small prospective series, as summarized in our review; however, mature, randomized phase III data comparing efficacy with RC are still lacking [50]. Second, as already mentioned, the question remains whether a gap exists between clinical and pCR post-NAC, following studies that prove discordance between clinical and pathologic staging in nearly half of cystectomized MIBC patients and occult lymph node metastases in approximately 5% of responding patients [16]. This poses a major challenge in adopting bladder-preserving strategies and raises concerns regarding the potential risk of undertreatment in patients harboring undetected residual or subclinical disease. Third, conventional staging with abdominopelvic CT scans is reported to be of limited value regarding response and nodal status assessment and for determining subsequent treatment after neoadjuvant systemic therapy [52]. These issues highlight the need for optimizing and standardizing the definition of cCR to render it a robust endpoint for further decision making and reliably selecting patients for bladder preservation. The integration of more sensitive imaging modalities together with emerging biomarkers may help reduce false-negative assessments of residual disease and improve the reliability of cCR as a surrogate marker for pCR.

In this context, there is growing interest in incorporating mpMRI for response assessment after NAC, together with cytology, cystoscopy with or without bladder biopsies, albeit current data are derived mainly from retrospective trials [53,54,55,56]. Preliminary findings from a study on Vesical Imaging-Reporting and Data System specialized for response assessment after NAC (NacVI-RADS) showed promising results in the ability of mpMRI to match final RC pathology with pre-surgical clinical stages, both for complete and partial responders [54]. Reported specificity and sensitivity of diffusion-weighted MRI for predicting pCR in MIBC patients exceeded 95%, ultimately aiding decision making between radical treatment, surveillance, or other bladder-sparing approaches [53,55]. A recent meta-analysis also evaluated the role of 18 F-FDG PET/CT for prediction of tumor response to NAC, demonstrating satisfactory diagnostic performance with a pooled sensitivity and specificity for prediction of CR of 0.94 (95% CI, 0.85–0.98) and 0.73 (95% CI, 0.42–0.91), respectively [57]. Nevertheless, further validation of these imaging modalities in prospective trials is awaited before their introduction in routine clinical practice.

Several ongoing trials attempt to further elucidate the potential value of cCR after neoadjuvant systemic treatment alongside supplementary biomarkers to guide subsequent decision making. The randomized phase II INSPIRE trial focuses on lymph-node-positive MIBC patients who have received ≥3 cycles of induction chemotherapy and are then randomized to undergo either CRT alone or CRT combined with durvalumab. The primary endpoint is cCR [58].

Finally, besides CRT, active surveillance is emerging as another bladder-preserving strategy after cCR with encouraging results [59,60,61]. In contrast to CRT, which aims to consolidate local control through definitive local treatment, active surveillance relies on close monitoring with the intent to defer or avoid radical therapy unless recurrence occurs. While CRT may offer improved local disease control by treating potential residual microscopic disease within the bladder, surveillance strategies aim to minimize treatment-related morbidity and preserve quality of life in carefully selected patients. However, the optimal selection criteria for each approach remain unclear, and the relative benefits and risks of surveillance versus CRT in patients with cCR have not yet been established.

The interim results of the single-arm phase II SURE-02 trial are robust, with 38.7% of MIBC patients achieving cCR, defined by negative MRI and reTURBT following neoadjuvant Sacituzumab-govitecan and pembrolizumab. Complete responders were allowed to forego RC, and all the patients were bladder-intact and event-free at 12 months, with acceptable grade 3 toxicity. Further molecular analysis revealed that a luminal and genomic unstable tumour profile was correlated with significantly increased cCR rates [62]. Following the RETAIN-1 trial [60], the RETAIN-2 trial recruited cT2-T3N0 MIBC patients who received three cycles of neoadjuvant nivolumab and dose-dense MVAC; patients underwent next-generation sequencing for detection of mutations in ATM, RB1 and ERCC2 genes and those with positive mutations and cCR (cT0) proceeded with active surveillance, whereas non-complete-responders were treated with RC, CRT or intravesical therapy. After a median follow-up of 22 months, results are quite promising for the surveillance group, with 60% of patients being free of metastases and having their bladder preserved [61]. The EORTC STARBURST-1 trial aims to develop and validate a tool for assessment of response to neoadjuvant systemic therapies, incorporating not only cystoscopy and mpMRI of the bladder, but also measurement of circulating plasma and urinary tumor DNA and urinary biomarkers. This tool is subsequently planned to be tested in the phase III STARBURST-2 trial, with the goal of personalizing treatment, with de-intensification in complete responders—omitting surgery—and escalation in non-responders [63]. Lastly, the ongoing multicenter phase II/III NEO-BLAST trial (NCT06537154) randomizes patients with non-metastatic MIBC who achieve cCR to neoadjuvant treatment—defined by negative urinary and circulating tumour DNA, MRI and TURBT—to either definitive treatment (RC or TMT) or active surveillance. The trial aims to investigate whether de-escalation to active surveillance is non-inferior to the standard of care—including chemoradiotherapy—in the responding subset of patients [64]. As no direct comparative data currently exist between active surveillance and CRT in this setting, the results of ongoing prospective trials may help clarify which patients may safely undergo surveillance and when consolidative CRT should be preferred.

## 5. Conclusions

This review summarizes the current evidence regarding bladder preservation with CRT in the subgroup of MIBC patients with response to neoadjuvant systemic therapy. Although several prospective and retrospective studies suggest that bladder-preserving strategies may be feasible and associated with encouraging oncologic outcomes in selected patients, the available evidence remains limited. Most published studies consist of small phase II trials or retrospective series, and direct randomized comparisons between RC and CRT in patients achieving cCR are lacking. Consequently, the current data should be interpreted cautiously, and bladder-preserving strategies following response to neoadjuvant therapy should still be considered investigational. Patients who benefit the most and experience high rates of cCR and favorable outcomes with respect to bladder-intact and OS rates are mainly those with T2, unifocal disease, with tumors amenable to maximal TURBT, without hydronephrosis and without concomitant CIS. Accurately matching cCR and pCR after neoadjuvant systemic therapy is still challenging. Novel systemic therapies, advanced imaging modalities and emerging biomarkers, including circulating tumor and urinary DNA, currently under investigation in ongoing trials, may enhance available response assessment tools, ultimately improving patient selection for bladder-preserving strategies and sparing patients with MIBC from unnecessary treatment escalation.

## Data Availability

No new data were created or analyzed in this study. Data sharing is not applicable to this article.
